# Nanophotonic biosensors harnessing van der Waals materials

**DOI:** 10.1038/s41467-021-23564-4

**Published:** 2021-06-22

**Authors:** Sang-Hyun Oh, Hatice Altug, Xiaojia Jin, Tony Low, Steven J. Koester, Aleksandar P. Ivanov, Joshua B. Edel, Phaedon Avouris, Michael S. Strano

**Affiliations:** 1 Department of Electrical and Computer Engineering, University of Minnesota, Minneapolis, MN USA; 2 Institute of Bioengineering, École Polytechnique Fédérale de Lausanne (EPFL), Lausanne, Switzerland; 3 Department of Chemical Engineering, Massachusetts Institute of Technology, Cambridge, MA USA; 4 Department of Chemistry, Imperial College London, London, UK; 5 IBM T. J. Watson Research Center, New York, NY USA

**Keywords:** Biosensors, Materials for optics, Nanoscience and technology, Optical properties and devices, Nanophotonics and plasmonics

## Abstract

Low-dimensional van der Waals (vdW) materials can harness tightly confined polaritonic waves to deliver unique advantages for nanophotonic biosensing. The reduced dimensionality of vdW materials, as in the case of two-dimensional graphene, can greatly enhance plasmonic field confinement, boosting sensitivity and efficiency compared to conventional nanophotonic devices that rely on surface plasmon resonance in metallic films. Furthermore, the reduction of dielectric screening in vdW materials enables electrostatic tunability of different polariton modes, including plasmons, excitons, and phonons. One-dimensional vdW materials, particularly single-walled carbon nanotubes, possess unique form factors with confined excitons to enable single-molecule detection as well as in vivo biosensing. We discuss basic sensing principles based on vdW materials, followed by technological challenges such as surface chemistry, integration, and toxicity. Finally, we highlight progress in harnessing vdW materials to demonstrate new sensing functionalities that are difficult to perform with conventional metal/dielectric sensors.

## Abstract (web summary)

This review presents an overview of scenarios where van der Waals (vdW) materials provide unique advantages for nanophotonic biosensing applications. The authors discuss basic sensing principles based on vdW materials, advantages of the reduced dimensionality as well as technological challenges.

## Introduction

Photonic biosensors are indispensable tools for life sciences and medical diagnostics. For example, commercial surface plasmon resonance (SPR)^1,2^ instruments are widely used to measure the binding kinetics and affinities of receptor–ligand interactions via the evanescent waves of surface plasmon polaritons (SPP) in a gold film. The real-time, label-free sensing capability of this modality has been critical for the functional and quantitative characterization of antibodies^3^, drugs, aptamers, target-drug interactions^4^, and virus-host-cell receptor interactions^5^. SPR generates invaluable time-resolved kinetics data that cannot be obtained with conventional end-point binding assays that employ target labelings, such as enzyme-linked immunosorbent assays (ELISA) or radioimmunoassays. State-of-the-art SPR instruments can achieve limits of detection (LOD) that are comparable to ELISA and they are increasingly being applied toward the clinical analysis of patient biofluids to detect proteins, microRNAs, drugs, and small molecules associated with various disease conditions^6^. The refractive index (RI)-sensing transduction mechanism of SPR eliminates the need for labeling and washing steps, provides real-time kinetic information and is fast, which can be advantageous for clinical applications. Furthermore, SPR is useful for characterizing low-affinity analytes, which in equilibrium ELISA assays would require larger sample volumes and cause unwanted dissociation of weakly-bound analytes during wash steps^6^.

To miniaturize and further improve the performance of SPR sensors, researchers have leveraged both top-down lithography and bottom-up synthesis to build “nanoplasmonic” sensors by engineering the flat gold films of conventional SPR into nanoparticles, nanoholes, or collections of sub-wavelength unit cells called “metasurfaces” of various shapes. Such nanostructures and metasurfaces can extend resonances to broader frequency ranges (i.e., from visible to near- or mid-IR), and exhibit optical phenomena such as localized surface plasmon resonance (LSPR), radiative coupling, and extraordinary optical transmission^7–9^. While nanoplasmonic structures add fabrication complexity and costs, they can improve multiplexing capacity, miniaturization, and sensitivity while also offering considerable design flexibility. Early demonstrations focused on enhancing the performance of conventional SPR by reducing the footprint of the sensing area to provide portability for point-of-care (POC) applications and/or increase parallel detection for high-throughput screening. For example, the widely used gold nanohole arrays^10,11^ can be excited with simple collinear optics compatible with a standard microscope, while simultaneously enabling array-based sensing via parallel imaging techniques^12^. The challenge of producing large-scale patterns with ease and low cost has been addressed using interference lithography^13^, replication from templates^14^, and photolithography^15^.

**Fig. 1:**
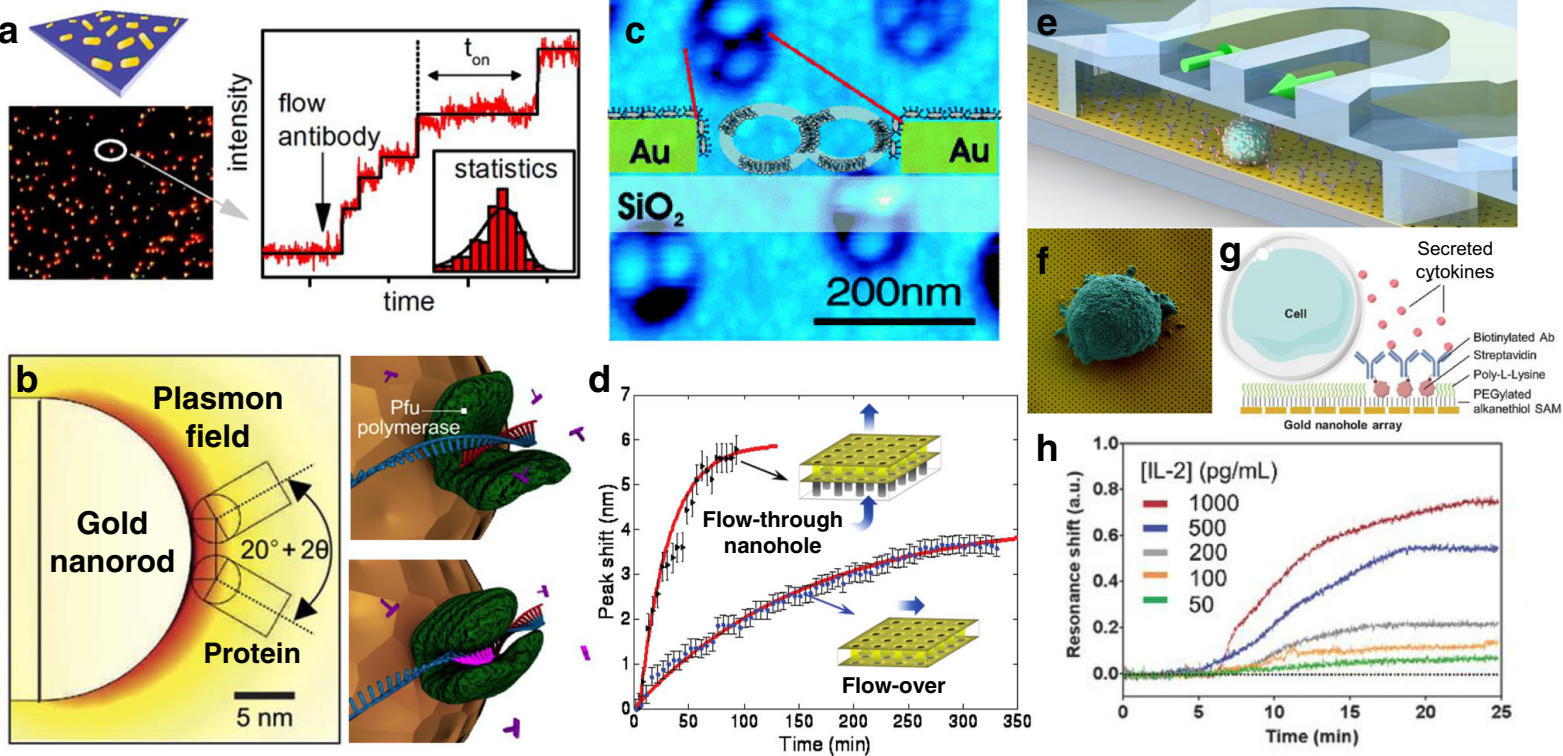
Specialized applications for metal nanoplasmonic sensors. **a** Real-time label-free plasmonic detection of single-molecule binding events. Wide-field imaging of hundreds of single gold nanorods enables multiplex single-molecule sensing. Reprinted from ref. ^17^ with permission. **b** Plasmonic gold nanorods coupled to a whispering gallery mode resonator were used to monitor DNA/polymerase interactions. Reprinted from ref. ^18^ with permission. **c** Lipid nanovesicles localized inside gold nanohole plasmonic sensors can be used for studying membrane-mediated biorecognition events. Reprinted from ref. ^11^ with permission. **d** Nanohole arrays can be used as nanofluidic channels. In this “flow-through” sensing scheme, the response time can be improved compared with the conventional “flow-over” approach. Adapted from ref. ^20^ with permission. **e** Gold nanohole array plasmonic sensor is integrated with microfluidics for in situ detection of cell-secreted molecules. **f** Electron micrograph of a single cell attached to the gold nanohole array. **g** Sideview schematic of the system. **h** Measured binding kinetics of cytokines secreted from a single cell inside a microfluidic chamber. Reprinted from ref. ^15^ with permission.

Nanoplasmonic sensors can perform tasks inaccessible to conventional SPR (Fig. 1). The main advantage of SPR compared to other diffraction-based optical techniques is the use of the tightly-confined evanescent field of SPP waves beyond the diffraction limit that is present at the interface of metal and medium. By shaping the flat metal films into nanoparticles, even tighter 3D field confinement is possible, and this eventually led to the ultimate feat in label-free sensing—detection of a single protein molecule (Fig. 1a, b)^16–18^. By taking advantage of the nanohole geometry, which can selectively localize receptor molecules or biological nanoparticles, researchers have shown unique sensing functions utilizing nanovesicles^11^ (Fig. 1c) and virus-like particles^19^. Nanohole SPR sensors integrated with sophisticated microfluidics have also been used to enhance molecular binding kinetics^20,21^ (Fig. 1d) or measure secreted molecules from a live single cell on a chip (Fig. 1e–h)^15^.

**Fig. 2:**
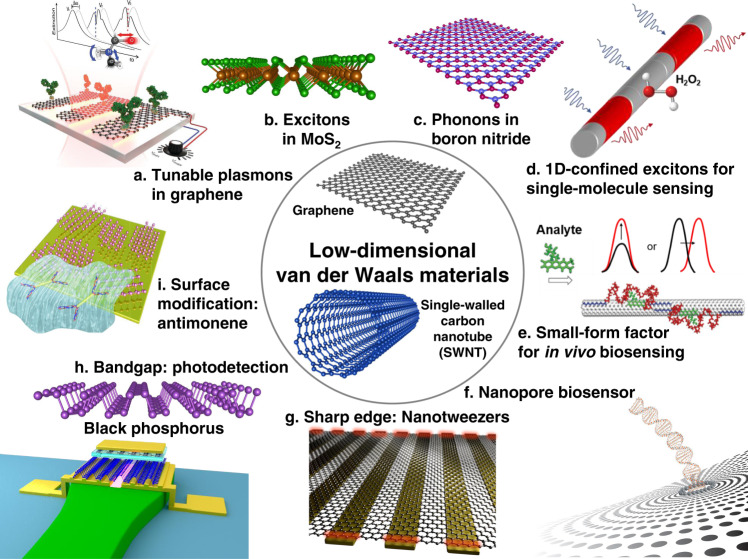
Low-dimensional vdW materials for nanophotonic sensing applications. Polaritons in 2D vdW materials can offer novel sensing modalities. **a** Plasmons in graphene (Adapted with permission from ref. ^26^). **b** Excitons in MoS_2_. **c** Phonons in boron nitride. 1D vdW materials (SWNT) have been used for (**d**), exciton-based single-molecule detection (adapted with permission from ref. ^27^) and (**e**) in vivo biosensing (adapted with permission from ref. ^28^). vdW materials can also be utilized to construct. **f** Single-molecule nanopore sensor. **g** High-gradient-field dielectrophoretic nanotweezers. **h** On-chip photodetector, and (**i**) high-affinity surfaces for specific analytes (adapted with permission from ref. ^31^).

More recently, a rapidly expanding family of van der Waals (vdW) materials^22,23^ with extraordinary optical, electrical, and mechanical properties have revolutionized the fields of electronics and optics. The reduced dimensionality of these materials enhances plasmonic field confinement, and their much-reduced dielectric screening confers sensitive electrostatic tunability and enables the excitation of different polariton modes such as plasmons, excitons, and phonons (Fig. 2a–c) for new sensing modalities^24–26^. While still a nascent technology relative to ELISA or SPR, two of the most extensively studied low-dimensional vdW structures—one-dimensional (1D) single-walled carbon nanotubes (SWNTs) and two-dimensional (2D) graphene—have already demonstrated novel sensing capabilities that are inaccessible to metal/dielectric nanophotonic sensors. For example, SWNTs have been used for single-molecule detection^27^ (Fig. 2d) and in vivo detection^28^ (Fig. 2e) via excitonic effects. 2D vdW materials such as graphene have been shown to increase plasmonic field confinement much tighter than metallic nanostructures. Graphene plasmonics has also demonstrated the unique potential for dynamically tunable infrared absorption spectroscopy for probing structural changes in molecules and vibrational-mode fingerprinting^26^. vdW materials could also enable the on-chip integration of electrical readouts, nanopore sensing^29^ (Fig. 2f), molecule trapping mechanisms (Fig. 2g), on-chip photodetectors^30^ (Fig. 2h), and nanofluidics. Hence, rather than merely competing for the same applications as existing modalities (e.g., refractometric sensing of receptor–ligand binding kinetics with metal-based SPR), we envision vdW nanophotonic sensors ushering in new capabilities for biosensing based on novel physical principles and materials properties^31^ (Fig. 2i).

In this review, we explore the potentials of vdW materials for nanophotonic biosensing (Fig. 2). We start by investigating performance metrics for nanophotonic sensors and then discuss how vdW materials can push their limits of performance, overcome tradeoffs, or enable new functionalities. We next discuss technological challenges awaiting researchers, including efficient excitation of high-momentum polaritons in vdW materials, nano-patterning, device architecture, surface chemistry, in vivo sensing, and toxicity. Finally, we highlight recent progress in harnessing vdW materials to demonstrate new modalities that are difficult to perform with conventional metal/dielectric nanophotonic sensors.

## Performance metrics for nanophotonic biosensors

How can one fairly evaluate the performance of various nanophotonic biosensors featuring different substrate materials, structures, transduction mechanisms, operating frequencies, and detection schemes? Consider SPR-based refractometric biosensing, in which signal transduction occurs via interfacial RI changes caused by surface-bound analytes. Ideally, such devices should provide resonance peaks or dips that undergo large spectral shifts upon analyte binding, are narrow as measured by the quality (*Q*) factor, and are of high contrast. When these conditions are met, analyte molecules can result in large intensity changes in the photodetector (Box 1).

As illustrated in Box 1, a single performance metric (e.g., spectral shift, *Q* factor, FOM, or resolution) may not be enough to characterize the LOD of disparate biosensors. A more fundamental quantity for nanophotonic structures is the evanescent field decay length of the resonant field into the sensing medium (Box 1f). The decay length is important not only for refractometric sensing but also for surface-enhanced Raman scattering (SERS) and surface-enhanced infrared absorption (SEIRA). Indeed, a marquee advantage of nanophotonics is the ability to beat the diffraction limit and confine optical energy into sub-wavelength scales. For instance, the decay length of the evanescent field into the dielectric medium of an SPP wave in a semi-infinite metal film is ~200 nm for water/metal interfaces at visible wavelengths. Decay lengths associated with LSPR in nano-patterned structures can be much shorter (i.e., tens of nanometers), allowing them to overcome the lower bulk sensitivity and detect thin films, or even an unlabeled single protein molecule. Near-field confinements in 2D vdW materials can be extreme: ~10^6^ smaller than the diffraction limit.^32^ In graphene nanoribbons, the plasmon field decays exponentially as ${\exp }\left(-\frac{\pi \alpha }{W}z\right)$, with $W$ being the width of the ribbon, where $\alpha \, \space {\rm{of}} \sim \!1$ is generally assumed but can approach $\sim \! 100$ at the edges of the graphene nanoribbon^33^. In a typical nanoribbon of $W= \, \sim \!100\text{nm}$, more than 50% of the plasmon intensity can be confined to a ~5 nm distance from the graphene surface. The practical LOD will depend on a combination of the field decay length and the sizes of the receptors and analytes^34,35^. Besides these intrinsic performance metrics, the real-world performance, and utility of nanophotonic biosensors depend on other factors such as surface modification and blocking strategies, multiplexing capacity, pre-concentration techniques, and signal-enhancing schemes.

Box 1. Operating principles and performance metrics for SPR biosensors In this illustrative example, receptor molecules (recombinant version of viral spike protein from (a) SARS-Cov and (b) SARS-Cov2) are immobilized on the gold SPR sensor surface and their binding interactions with the target analytes (human ACE2 protein) are measured at different concentrations. A standard equation for 1:1 binding kinetics calculates the change in the normalized surface coverage, *c*(t), of bound receptor-analyte pairs by subtracting the rate of dissociation from the rate of association:

$$\frac{\partial c(t)}{\partial t}={r}_{\mathrm{{association}}}-{r}_{\mathrm{{dissociation}}}={k}_{\mathrm{{on}}}{c}_{\mathrm{a}}(1-c(t))-{k}_{\mathrm{{off}}}c(t),$$

where *c*_a_ is the analyte concentration in solution, *k*_on_ (mol^−1^ L s^−1^) is the association rate and *k*_off_ is the dissociation rate. The equilibrium dissociation constant *K*_d_ = *k*_off_/*k*_on_ (M) measures binding affinity (lower *K*_d_ means higher binding strength) and the equilibrium surface coverage is ${c}_{\mathrm{{eq}}}/{c}_{{\max }}=1/(1+{K}_{D}/{c}_{a})$. In this example (Table c), SARS-CoV-2 (2019) shows higher affinity (lower *K*_d_) than SARS-CoV (a and b were plotted using the 1:1 binding kinetics equation and parameters from ref. ^5^), providing quantitative clues to its more infectious nature. (d) An example of reflectance vs. wavelength obtained from a conventional prism-based SPR setup. Conventional prism-based SPR has relatively broad resonance and typically the quality-factor (Q; resonance wavelength divided by resonance linewidth) is on the order of 10. Resonance shift in SPR is measured by either bulk sensitivity (shift per RI change of the bulk solution), or the more biologically relevant thin-film sensitivity (shift per adsorbed film thickness). Conventional prism-based SPR setups exhibit very high bulk sensitivity of up to ~10,000 nm/RIU, or ~100°/RIU^2^. Resolution is defined as the minimum detectable RI change. This depends on multiple factors such as instrumentation technique, resonator design, and environmental control^156^. Despite relatively low Q’s, with external controls, robust curve-fitting algorithms, and schemes to reduce photodetector shot noise and source fluctuation, state-of-the-art SPR can resolve spectral shifts as small as 0.001 nm from a resonance dip^156^, which equates to a resolution of (0.001 nm)/(10,000 nm/RIU)/ = 10^−7^ RIU. However, bulk sensitivity does not always correlate with sensitivity to the molecular binding. For example, while nanoplasmonic sensors (e.g., the gold nanohole sensors^157^) (e) typically show lower bulk sensitivity (~700 nm/RIU in this example) than conventional SPR, they utilize more tightly localized evanescent fields and could show similar performance for detecting surface-bound molecules. Conversely, long-range SPPs show higher bulk sensitivity (~60,000 nm/RIU)^158^ than conventional SPR, but their molecular LOD is similar. A good sensitivity metric for surface-bound molecules can be estimated in terms of picograms/cm^2^, and thin-film sensitivity (shown in (f)), as measured by resonance shifts with deposited Al_2_O_3_ film thickness on gold nanodisks/nanoholes^34^ is a practical parameter for predicting detection limits^159^. Using new degrees of freedom available with nanostructures, researchers have engineered high-Q resonances. The figure-of-merit (FOM), defined as (bulk sensitivity)/(linewidth) considers intrinsic/extrinsic plasmon damping mechanisms and can serve as another useful metric. In particular, when an intensity-based sensing scheme is used (e.g., SPR imaging), a narrower peak results in a greater intensity change at a fixed wavelength per RI change. The FOM can be increased by engineering structural resonances (e.g., Fano resonance or dark-mode^157,160^) to reduce the resonant linewidths. However, such changes also tend to reduce the intensity of the resonance, thereby degrading the overall sensitivity of detection. Likewise, arguments based on Q or FOM alone would favor high-Q dielectric resonators over metal plasmonics, but their molecular detection sensitivities are relatively similar. This is due in part to the low contrast in signal, determined by the relative magnitude of the resonant peaks. For continuous monitoring of binding kinetics at high temporal resolution (~millisecond acquisition time), it is essential to deliver high photon flux at the photodetector, which requires that the resonance dip or peak should be large in magnitude (i.e., high contrast).
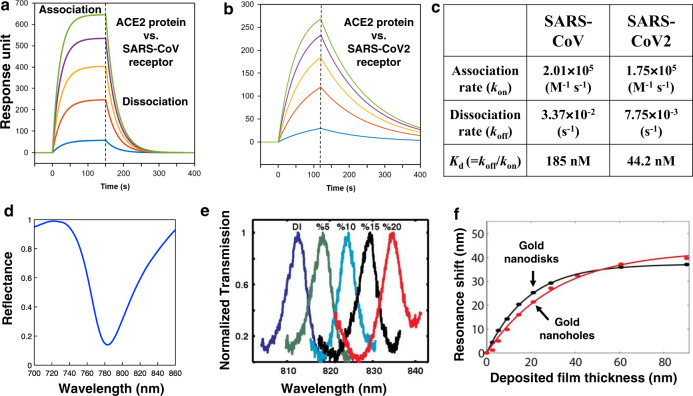

Illustrative examples of SPR binding kinetics for purified recombinant receptor-binding domains of viral spike protein from (a) SARS-CoV and (b) SARS-CoV2, against human ACE2 protein plotted using (c) association and dissociation rates from ref. ^5^ and the 1:1 binding kinetics equation. (d) Example of SPR reflection spectrum from a prism-based setup. (e) Resonance shift measured for a high-quality-factor subradiant SPP mode with changing NaCl concentration. Reprinted with permission from ref. ^157^. (f) The resonance shift vs. ALD-grown Al_2_O_3_ film thickness was measured from which one can obtain the LSPR field decay length. Adapted with permission from ref. ^34^.

## VdW materials for enhancing conventional prism-coupled SPR biosensors

Building on the mature capabilities of prism-coupled gold-film SPR instruments, researchers have explored the benefits of coating the gold film with various 2D materials. Groups have reported improved SPR sensitivity from graphene-coated gold surfaces, which was attributed to enhancement of the surface electric fields provided by an added charge transferred from graphene to gold^36,37^. While the surface of gold SPR sensors is commonly functionalized via a self-assembled monolayer of alkanethiols, the surface of graphene or other vdW materials requires different strategies for functionalization, which presents challenges as well as new opportunities. Graphene surfaces can be modified by attaching some molecules via π-stacking or other functional groups (e.g., carboxyl). However, the ease of attachment via π-stacking also implies increased nonspecific binding of interferents from biological samples, necessitating a proper blocking procedure. Xue et al. used antimonene-modified gold SPR chips to detect microRNA hybridization events^31^. Density functional theory energetic calculations predicted that antimonene surfaces have a higher affinity for single-stranded (ss) DNA than double-stranded (ds) DNA. By using gold nanorods to further boost SPR signals, they were able to detect ssDNA-microRNA hybridization events. vdW materials can also protect the surfaces of reactive metals—especially copper and silver—which is necessary for SPR biosensing applications^38^. Wu et al. characterized the potential of layer materials (e.g., graphene, boron nitride, and carbon nanomembranes) to protect the chemical and optical properties of plasmonic metals^39^ At a wavelength of ~1 µm, they achieved bulk RI sensitivity of ~10,000 nm/RIU, which is comparable to the sensitivity of conventional spectroscopic techniques.

While these studies demonstrate impressive LOD for molecules in buffer, the response of hybrid vdW-metal surfaces in biofluids for practical biosensing applications (e.g., plasma) needs to be characterized to ensure whether the same low LOD can be maintained. Also, instead of single-channel measurements, reference-channel measurements, as well as temperature stabilization, will be essential for ultrasensitive detection.

As shown in these examples, the applications of vdW materials for conventional SPR or other nanophotonic RI sensors are rapidly expanding^40^. However, the large-area growth and transfer of vdW materials atop metal films add complexity, labor, and costs. As such, in order to justify their use in practical biosensing applications, the benefits need to go beyond incremental gains in sensitivity and enhanced surface adsorption of analytes. Ideally, vdW nanophotonic biosensors should be used in tandem with other sensing modalities, such as electrical or electrochemical detection, or to enable novel functionalities such as vibrational fingerprinting with spectroscopy.

## VdW materials for reconfigurable vibrational spectroscopy

Refractometric sensors made with metals, dielectrics, and metal-vdW hybrids have shown impressive performance metrics, but cannot identify bound analyte molecules. Vibrational spectroscopy techniques like infrared (IR) absorption spectroscopy and Raman scattering complement SPR and enable such “fingerprinting” functions for molecules, viruses, extracellular vesicles, and cells. With their electrically tunable doping and ultra confined mid-IR and terahertz (THz) plasmons, graphene and other 2D vdW materials are especially promising for these applications.

IR absorption spectroscopy is a powerful analytical tool due to its ability to reveal molecular and structural information of samples without using external labels. Samples in solid, liquid, and gas phases have distinct vibrational modes associated with their molecular bonds, particularly within the mid-IR spectrum of ~3–20 μm (3000–600 cm^−1^). IR spectroscopy taps into this so-called fingerprint region and is used in numerous scientific fields (e.g., biology, chemistry, material science) and in the industry (e.g., food safety, pharmacology, environmental monitoring, and forensics). However, the large mismatch between micrometer IR wavelengths and nanometer-sized molecules leads to low sensitivity, limiting the use of IR spectroscopy for measuring trace amounts of samples. Nanophotonics can bridge this gap by producing strong, tightly localized near-fields in the vicinity of resonant optical nanostructures. This approach is called SEIRA (Box 2)^41–44^, and is closely related to SERS. VdW materials have been used to enhance SERS in the absence of plasmon excitation. The effect is not direct but occurs via chemical enhancement of the polarizability of graphene-bound molecules^45,46^. We focus here on mid-IR SEIRA enabled by the plasmons of vdW materials.

**Fig. 3:**
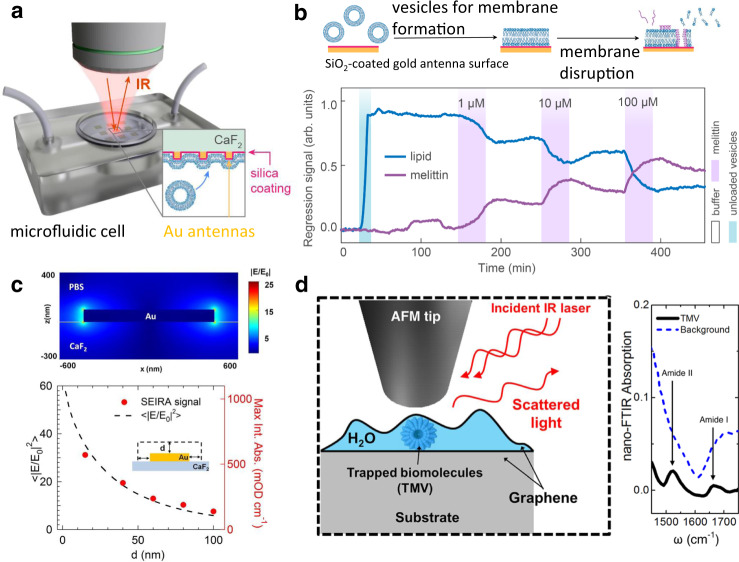
Detecting biomolecules with mid-IR spectroscopy. **a** Reflection-mode in situ SEIRA detection of multiple biological analytes. **b** Time-resolved and molecule-specific in situ SEIRA monitoring of the lipid membrane formation and disruption dynamics. Lipid vesicles rupture on SiO_2_-coated gold antennas and form a supported lipid bilayer membrane. Subsequent injection of cytolytic peptide, melittin, forms pores and disrupts the membrane. **a**, **b** Adapted from ref. ^47^ with permission. **c** Calculated electric field enhancement for a gold nanotenna in a buffer. Distance dependence of SEIRA signal comparing computer simulations (dotted) and measured absorbance data (full dots). Reprinted from ref. ^48^ with permission. **d** Graphene liquid cell for detection of biomolecules trapped in a thin layer of water. Reprinted from ref. ^50^ with permission.

Progress in nanophotonics has fueled research into increasing low IR signals for SEIRA and expanding its application space. Resonantly excited plasmons in SEIRA substrates (e.g., nanorods or nanogaps) typically exhibit lower enhancement factors (EF) of ~10^3^–10^7^ but provide a wider probing range extending a few hundred nanometers from the metal surface, which is suitable for sensing adsorption of large protein molecules, binding of surface-linked receptors and target molecules, biomembranes, or nanovesicles. These benefits are illustrated in recent experiments (Fig. 3a, b), which detected the kinetics of multiple biological analytes in aqueous solutions using resonant gold nanorods coated with a thin SiO_2_ layer to facilitate the formation of biomembranes^47^. Limaj et al. characterized the distance dependence of SEIRA EF using SiO_2_ overlayers of different thicknesses (Fig. 3c). They showed that the absorption signal from molecules was detectable for SiO_2_ overlayer thicknesses—and hence, metal-to-molecule separation distance—of up to ~100 nm in aqueous solutions^48^. This distance dependence is consistent with the dry characterization of gold antennas for SEIRA^49^. Although SERS has much shorter probing ranges of just a few nm, the longer range of SEIRA is advantageous (Box 2) by allowing the use of receptor molecules for specificity as well as to detect larger analytes (e.g., biomacromolecules, extracellular vesicles, and viruses). Khatip et al. utilized graphene to trap a thin layer of sample solution for tip-enhanced IR spectroscopy in an aqueous environment^50^ (Fig. 3d).

To date, SEIRA has mostly employed metal-based plasmonic resonators with engineered nanostructures in various shapes and array configurations. However, this landscape is changing rapidly, with novel nanomaterials that can address the intrinsic shortcomings of metals^51^. For instance, while metals can support strong plasmonic responses due to their high electron concentration, they suffer from high loss and provide resonators with low Q-factors (typically < 10). In contrast, Mie resonances in low-loss and high-index dielectric metasurfaces can achieve several orders of magnitude higher Q-factors. Tittl et al. used CMOS-compatible high-Q-factor (>100) silicon resonators in a sensing approach that can convert mid-IR molecular fingerprints into chemical-specific barcode-like images while eliminating the need for bulky IR spectroscopy instruments^52^. Leitis et al. used germanium metasurfaces to expand the spectral coverage of resonant SEIRA over 1000 cm^−1^ wavenumber and extracted vibrational signatures of different molecules in a multi-step assay^53^.

**Fig. 4:**
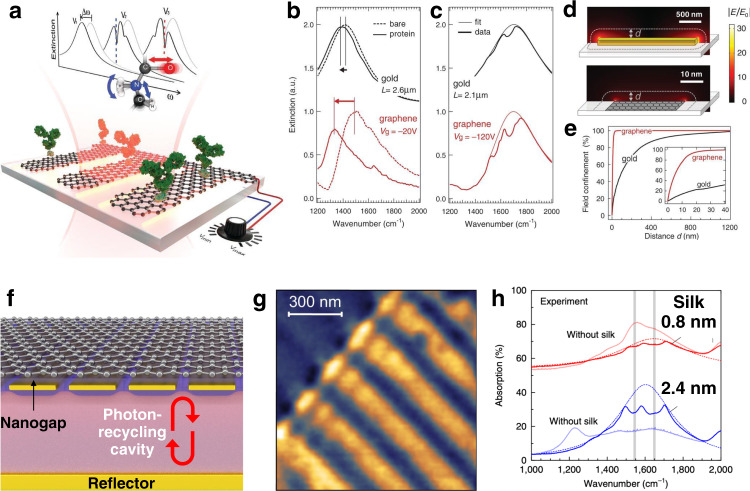
Graphene-based SEIRA strategies. **a**–**e** Graphene-based mid-IR plasmonic biosensing. Adapted from ref. ^26^ with permission. **f** Tightly confined graphene acoustic plasmons for sensing a thin layer of protein molecules. Reprinted from ref. ^54^ with permission. **g** Near-field of graphene acoustic plasmons propagating along with an array of gold ribbons (bright regions). Adapted from ref. ^66^ with permission. **h** Tightly confined graphene acoustic plasmons can detect a thin layer of protein molecules with high sensitivity. Reprinted from ref. ^54^ with permission.

Graphene and other 2D vdW materials are also bringing exciting prospects for SEIRA^26,46,54^. The unique optoelectronic properties of graphene enable external control over plasmonic resonance frequency by changing its carrier density (i.e., Fermi level) with electrostatic biasing^55–57^. Such active tuning is used to realize dynamically configurable IR sensors tuned to specific molecular vibrational modes. Rodrigo et al. showed the first graphene-based tunable mid-IR biosensor and its potential for quantitative chemical-specific detection^26^. They measured plasmon resonance spectral shifts accompanied by narrow dips corresponding to the surface-enhanced molecular vibration bands of a protein analyte over a broad spectrum (Fig. 4a–e). The plasmonic resonance is electrostatically tuned to sweep continuously over the two protein vibrational bands, amide-I and amide-II. Based on this dynamic tunability, they combined both SEIRA and LSPR sensing in a single device and showed extraction of complex refractive indices of nanometer-thick samples on the sensor surface. Since then, graphene-based SEIRA has been used for sensing biomolecules, polymers, self-assembled monolayers, ion gels, metal ions, and gases^54,58–61^.

Another unique feature of 2D vdW materials is their extreme field confinement. Given the semi-metallic nature of these 2D materials and the fact that their thickness can be close to atomic-layer scale, they can confine near-fields at extreme (i.e., ~10^6^-fold smaller than the diffraction limit). Graphene nanoribbons can provide up to two orders of magnitude higher field confinement than metallic plasmonic dipole antennas, and thus enable unprecedentedly strong overlap of light with nanometric biosamples^26^ (Fig. 4d, e). This feature is attractive for achieving superior sensing performance, especially for small molecule detection. Consequently, graphene nanoribbons can also be exploited to detect thin layers of adsorbed gas molecules, as recently demonstrated in a pressure-controlled chamber for label-free identification of various gases including SO_2_.^62^. Even tighter field confinement is possible by coupling graphene plasmons with their image charges in a metal mirror and forming a hybridized mode called acoustic graphene plasmons^54,63–66^ (Box 3). In this mode, the field energy is confined in a nanometric gap between graphene and mirror, thus sample insertion into the gap remains a practical challenge.

Graphene can achieve high Q-factors, which can lead to increased sensitivity due to longer interaction time between the probing IR light and the analytes. In theory, Q-factors well over 100 can be expected with exfoliated graphene^32^. These values favorably compare to metal-based resonators, which exhibit Q-factors of ~10 at similar frequencies^41^. Experimental Q-factors of graphene plasmons are far lower, however—typically <10. This is because chemical vapor deposition (CVD) is typically used to synthesize large-area graphene, and the resulting grain boundaries, impurities and defects result in lower quality graphene, as shown by its reduced carrier mobility (~1000 cm^2^ V^−1^ s^−1^). Furthermore, nanopatterning of graphene with small structures (e.g., nanoribbons with widths ~50 nm) is often required to excite mid-IR plasmons, but the rough edges induced by the nanofabrication process lead to additional loss and further deteriorate the quality of the resonators. Limited plasmon lifetimes (~50 fs) and inelastic scattering with optical phonons have also been reported in graphene nanoribbons^67^. However, further effort is needed towards the development of high-quality 2D vdW materials that can be scalably manufactured for real-world applications. Observation of low-loss graphene from the encapsulation of exfoliated samples with hexagonal boron nitride (hBN)^68^ and recent advances in CVD graphene transfer involving proper cleaning and encapsulation, which result in room-temperature mobility in excess of 10,000 cm^2^ V^−1^ s^−1^, demonstrate some of the important ongoing progress in materials development^69^.

Dynamic tuning of graphene plasmons (see Box 3) is achieved mainly by applying an external voltage (hence controlling the Fermi energy *E*_*f*_), but this requires some care in terms of fabrication and design. In the past, devices typically relied on a thick dielectric spacer between the graphene and the gating layer. This leads to low capacitance values, and hence requires the application of high bias voltages (tens of volts). However, recent research shows that incorporation of a field-effect transistor (FET) device architecture and suitable spacer films as a substrate can greatly reduce the bias voltage while enabling high-speed operation and incorporation of imaging^70^. A related issue stems from the dependence of the graphene response on the optical properties of the spacer layer. Graphene plasmons can strongly couple with the phonon modes of the substrate, and this hybridization can produce complex spectral features and limit the operating spectral window. Hu et al. incorporated CaF_2_ nanofilms as spacers; since CaF_2_ has no phonons in the relevant range, it eliminates this problem and allows the use of SEIRA over a broader spectral range^59^.

Although extreme light confinement with 2D materials is an attractive feature for sensing, it is characterized by weak coupling efficiency of the external light to graphene plasmons. The extinction values are typically quite low (below 5%), which is not practical for device applications. The coupling efficiency is further hampered by the low carrier density and the weak oscillator strength of graphene plasmons. In order to excite stronger plasmonic responses, recent works have used multi-layer stacks^71^, integration with photonic cavities (i.e., Fabry-Perot)^72^, and hybrid substrates incorporating plasmonic nanostructures^73^ (Fig. 4f–h). Strong field confinement also requires the presence of samples directly at the sensing surface, and in this context, graphene’s high adsorptivity for hydrophobic species can be leveraged to concentrate analytes. Functional polymers and active analyte transport schemes can also be utilized to enrich samples at the sensing layer to boost both signal strength and device response time. Active trapping methods, e.g., dielectrophoresis (DEP; Box 4) can also be implemented with vdW materials.

To benefit from these additional functions, graphene-based SEIRA devices need to be compatible with in-solution measurements and microfluidics. To this date, most SEIRA experiments have been conducted in a dry medium due to the high absorption of water and polymers used for microfluidics in the IR spectrum. These constraints are circumvented for metal-based SEIRA by utilizing strong plasmonic confinement and performing plasmon excitation and signal collection in the reflection configuration with an IR-transparent substrate^74^. Because the field intensity decays exponentially from the plasmonic substrate surface with a decay length of ~100 nm, one can selectively probe surface-adsorbed molecules and minimize interference from water molecules in the sample. In contrast, graphene resonances are extracted from either the extinction spectra in the transmission configuration or the absorption spectra obtained in the reflection configuration with an optically thick metal layer, which make in situ measurements not possible. Recent work has combined graphene with attenuated total reflection (ATR) prisms for in situ studies, but these approaches involve inherently bulky instrumentation and are not suitable to exploit tunable and strongly confined plasmonic resonances^75,76^. Graphene and other liquid-impermeable vdW materials offer alternative routes to avoid water absorption by trapping water in nanometric volumes. This approach has been successfully demonstrated with tip-enhanced IR absorption spectroscopy using a graphene liquid cell^50^, and in graphene-based nanofluidics^77^.

Besides graphene plasmons, phonon-polaritons in hBN have been utilized for confining mid-IR light^78–81^. The resonances of hBN, however, are spectrally limited in biosensing applications due to the fixed Reststrahlen bands—where the energy band between the longitudinal and transverse optic phonon frequencies Re(ε) is negative and surface phonon polaritons can be excited—which are not aligned with the amide absorption bands of proteins. However, recent work has shown that the Reststrahlen band of MoO_3_ can be tuned^82^, which may be useful for chemical sensing applications, although such tuning is not suitable for biomolecules such as proteins and nucleic acids.

Box 2. Surface-enhanced infrared absorption spectroscopy (SEIRA)

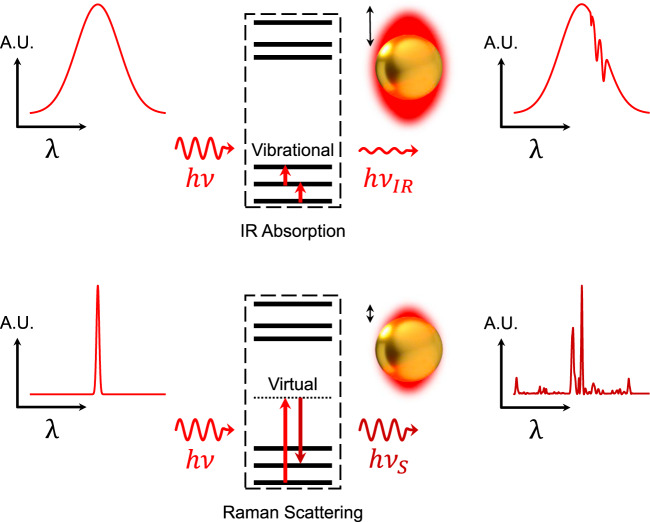
Raman scattering and IR absorption are commonly used to obtain molecular structural information. Raman spectroscopy is based on the inelastic scattering through which a molecule either gains energy from (Stokes process) or loses energy to (anti-Stokes process) incident photons. Transitions involved in IR absorption versus Raman scattering processes are subject to different selection rules. Thus, the measured spectra show different and complementary sets of peaks, and their utility depends on the specific application.

Raman scattering is extremely weak, as only 1 in 10^6^–10^10^ photons striking a molecule are scattered inelastically. Typical non-resonant Raman scattering cross-sections (*σ*_Raman_) are 10^−31^~10^−29^ cm^2^/molecule—much smaller than for IR absorption (~10^−20^ cm^2^/molecule). However, a key advantage of Raman spectroscopy for biosensing is the small scattering cross-section of the water, which minimizes unwanted background in aqueous samples. In contrast, absorption of mid-IR radiation in water can obscure absorption signals from analytes.

The scaling factor for electromagnetic enhancement for SERS as a function of electromagnetic near-field amplitude |*E*| is roughly |*E*|^4^, plus a chemical EF of 10–100-fold due to the modified polarizability of molecules on a metal surface, whereas SEIRA increases only with |*E*|^2^. In most SERS substrates, the spatial distribution of intense “hotspots” are random, thus the location of these spots cannot be easily predicted for continuous activation. In contrast, SEIRA EFs, while lower, can be more uniformly distributed and predictable. Importantly, the utility of SERS diminishes for probing large biomacromolecules or complexes, because SERS EF drops rapidly over a few nanometers from the metal surface. In addition, intrinsic SERS detection of protein molecules or changes in their orientation/conformation is difficult for large proteins. Autofluorescence can also easily mask weak Raman signals. Resonantly-excited plasmons in SEIRA substrates typically exhibit lower EFs but provide a wider probing range suitable for sensing the adsorption of larger targets.

The performance of a SEIRA substrate is represented by the EF, which measures the ratio of absorption from a substrate to that of a reference sample when normalized to an effective volume. This performance metric is given as:

$${\rm{SEIRA}}\,{\rm{EF}}=\left(\tfrac{{A}_{Dev}}{{V}_{Dev}}\right)/\left(\tfrac{{A}_{0}}{{V}_{0}}\right)$$

where *A*_*Dev*_ and *A*_*0*_ denote measured optical absorption from a SEIRA substrate and reference device, respectively, with *V*_Dev_ and *V*_0_ being the effective volumes where optical fields and target molecules overlap. Infrared reflection-absorption spectroscopy (IRRAS) is a common method to obtain reference signals from a thin film of target biomolecules. In this case, SEIRA EF can be rewritten as:

$${\rm{SEIRA}}\,{\rm{EF}}=\left(\tfrac{{A}_{Dev}}{{V}_{Dev}}\right)\times \left(\tfrac{{A}_{0}}{{V}_{0}}\right)\times \left(\tfrac{{\sin }^{2}\theta }{{\cos }^{2}\theta }\right)\times 2$$

where *A*_IRRAS_ and *θ* are measured absorption from IRRAS and the incident angle of IR radiation, respectively. The oblique incidence of IR radiation and the dipole-mirror effect is accounted for by $\frac{{{\sin }}^{2}\theta }{{\cos }\theta }\times 2$. Since *A*_Dev_ is calculated from the power dissipation by integrating $\frac{1}{2}\omega {\mathfrak{I}}\left({\varepsilon }_{{\rm{r}}}\right){\left|{E}_{{ex}}\right|}^{2}$ over the effective volume, where ω and ${\mathfrak{I}}\left({\varepsilon }_{{\rm{r}}}\right)$ are the angular frequency and the imaginary part of permittivity for an analyte, SEIRA EF is dependent on ${\left|{E}_{{ex}}\right|}^{2}$. To obtain a large SEIRA EF, therefore, a SEIRA substrate should be able to concentrate as much IR radiation as possible into a very small volume to maximize the absorption-volume ratio, which in turn increases the intensity of electric fields.

Box 3. Characteristics of tunable graphene plasmons

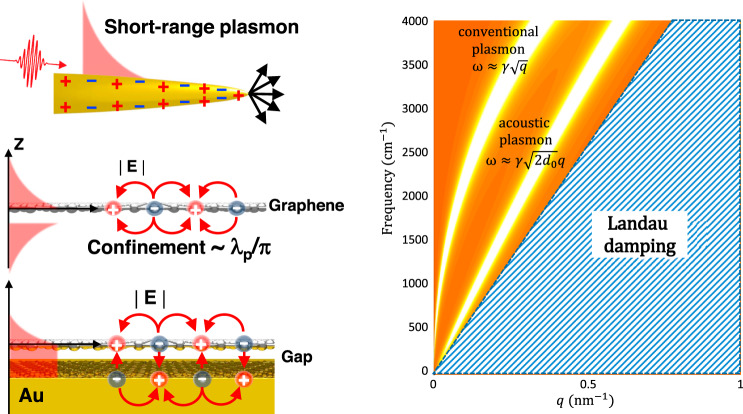
Graphene plasmons offer the unique advantages of tight field confinement as well as electrical tunability of plasmon resonances. Plasmons in 2D vdW materials represent the ultimate limit in terms of sub-diffraction field confinement. As shown in the left panel, graphene plasmons can be conceptualized as an extension of short-range SPP waves in a metal film with symmetric charges across the film, and these plasmons are further squeezed as the film is thinned down. The dispersion of conventional graphene plasmons scales as $\omega \approx \gamma \sqrt{q}$ in the quasi-static limit, where $\gamma \equiv \sqrt{{E}_{F}/{\varepsilon }_{{eff}}}$, *E*_*F*_ is the graphene Fermi level, *ε*_*eff*_ is the effective dielectric constant of the environment, and *q* is the momentum. By placing graphene in proximity to a metal plate, mirror image charges are created that modify the plasmon field distribution. The dispersion for the resulting acoustic graphene plasmon now becomes linear (i.e., $\omega \approx \gamma \sqrt{2g}\cdot q$), to leading order in the distance to the metal, ${g}$, and in the limit where ${v}_{F}q\ll \omega \ ({v}_{F}{:Fermi \ velocity})$.

The right panel shows conventional and acoustic graphene plasmon dispersion. In the context of nanoribbon plasmonic resonators, the plasmon momentum is related to the ribbon width, ${W}_{0}={\pi}/{q}$, which accommodates the plasmon half-wavelength. For the same graphene nanoribbon, the conventional plasmon resonances are higher than for their acoustic counterparts. Although the stiffness ${\gamma}$ of the plasmon resonance can be tuned with doping and dielectric environment, the plasmon must reside outside of the Landau damping region in order to retain its coherence (i.e., quality factor).

These dispersions assume the same graphene doping with *E*_*F*_ = −0.5 eV, which can be obtained with an out-of-plane electric field of 1 GV/m (i.e., the breakdown field for silica). On the other hand, the plasmon resonances of metallic nanostructures are fixed upon fabrication due to the high carrier concentration (typically 1 per atom) for noble metals. The 2D semi-metallic nature of graphene allows for electrical tunability that is not possible with conventional metals. Free carriers in graphene obtained through electrical doping can number 0.001–0.01 per atom, and the corresponding graphene plasmon resonance resides in the mid-IR (3–20 µm wavelength) and THz frequency ranges, which are ideally suited for reconfigurable vibrational spectroscopy with dynamically tunable resonances^24,26^.

Box 4. vdW materials for trapping of biomolecules via DEP Rapid transport of target analytes to the nanophotonic sensor is critical for detecting low-concentration analytes. Nanophotonic sensors typically exhibit small detection areas, and active transport and concentration of analytes can greatly improve the detection speed and overall sensitivity.^161^ DEP, a radiofrequency (kHz–MHz) analog of the optical trapping process, has been used to manipulate biological particles and can be useful in the context of vdW materials. With a relatively simple apparatus (electrodes and a function generator), DEP can be employed to attract, trap, or repel neutral particles based upon their polarizability. When a particle of radius *R* and complex permittivity ${\varepsilon }_{{\rm{p}}}^{\ast }(\omega )$ is in a medium of permittivity ${\varepsilon }_{{\rm{m}}}^{\ast }(\omega )$, the time-averaged gradient DEP force acting on it is approximated as^162^.

$${{\boldsymbol{F}}}_{DEP}=\pi {\varepsilon }_{m}{R}^{3}\cdot {\mathrm{Re}}\left(\frac{{\varepsilon }_{p}^{\ast }(\omega )-{\varepsilon }_{m}^{\ast }(\omega )}{{\varepsilon }_{p}^{\ast }(\omega )+2{\varepsilon }_{m}^{\ast }(\omega )}\right)\nabla {|{{\boldsymbol{E}}}|}^{2}$$

where $\left|{\boldsymbol{E}}\right|$ is the electric field strength. Since ${{\boldsymbol{F}}}_{{DEP}}$ scales as ~1/*dx*, it is possible to boost its magnitude by shrinking the critical dimensions of the system (e.g., electrode gap width or sharpness) without using high bias voltages or electric fields that are detrimental to biological systems (a). Sharp tips^163^, nanometric gaps, or nanopores^164^ in metal films have been used for a wide range of DEP applications such as particle filtering, sorting, trapping, levitation, and rotation^162^. Atomically sharp vdW materials are uniquely capable of creating ultra-strong DEP forces in the context of (b) sharp electrode tips realized with SWNTs^165,166^ and (c) graphene-edge DEP^167–169^. Barik et al. showed that patterned edges of monolayer graphene can generate high-gradient fields to trap nanoscale objects and molecules with enhanced trapping forces^168^. Such edge-trapping DEP devices can be made from graphene or other conductive vdW materials patterned over a metal electrode, separated by a thin insulator. Using this setup, polystyrene particles were trapped on the edges of graphene at voltages as low as 0.5 V. Furthermore, both long (10 kb) and short (500 bp) DNA strands were trappable at low voltages (~2–3 V).

The ultra-strong gradient forces at nanoscale distances in graphene-edge DEP have the potential for nanophotonic as well as nanoelectronic biosensing applications, where analytes such as antibodies, proteins, viruses, nanoparticles, or cells could be trapped rapidly using relatively low (~1 V) bias voltages. Furthermore, the electrical readout capabilities of some vdW materials could also enable a path toward hybrid optical/electrical DEP-enhanced sensors. Surface functionalization of the sensor could be utilized to further enhance detection selectivity. DEP can also be combined with rapidly advancing nanopore sensing technologies (d) based on vdW materials, as discussed in the Supplementary Information.

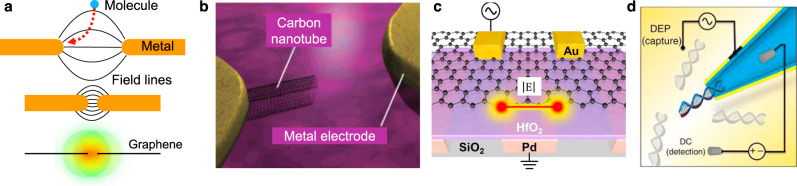
(a) Dielectrophoresis is a scalable particle trapping mechanism. Smaller electrode separation and sharper electrode tip can boost field gradients. (b) Carbon nanotube electrodes (adapted from ref. ^166^ with permission), (c) graphene edge electrodes (adapted with permission from ref. ^168^), and (d), metallic nanopores (adapted from ref. ^164^ with permission), can generate high gradient fields at relatively low trapping voltages toward trapping nanoparticles and biomolecules.

## VdW materials as building blocks for waveguide-integrated nanophotonic sensors

**Fig. 5:**
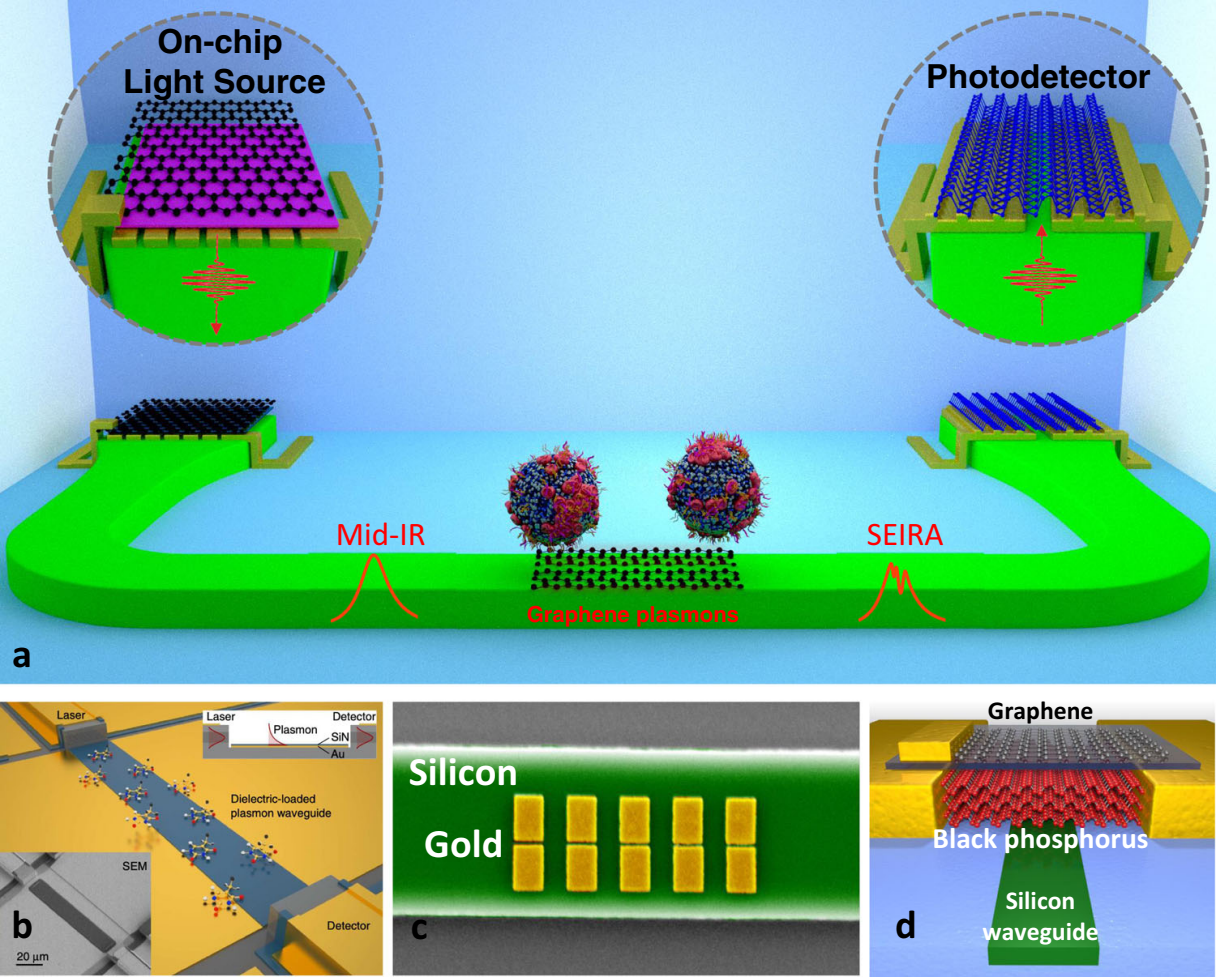
Progress towards lab-on-a-chip detector systems. **a** Illustration of a waveguide-integrated hybrid nanophotonic biosensing platform. By harnessing excitons, plasmons, and phonon-polaritons in a wide range of vdW materials, we envision that researchers will be able to integrate light sources, mid-IR sensors, nano-tweezers, and detectors on a chip. Image credit: Daehan Yoo. **b** Monolithically integrated lab-on-a-chip chemical sensing platform based on a quantum cascade laser and detection coupled with a gold plasmonic waveguide. Reprinted from ref. ^84^ with permission. **c** Waveguide-integrated SEIRA sensor. Five pairs of gold nanorods with a 30-nm gap were patterned on a silicon waveguide for SEIRA sensing of molecular monolayers. Reprinted from ref. ^85^ with permission. **d** Waveguide-integrated black phosphorus photodetector.

Tightly-confined excitons in graphene and other vdW materials offer additional exciting opportunities for nanophotonic biosensing. One long-term vision shared by many researchers is to realize compact nanophotonic “lab-on-a-chip” devices that integrate light sources, low-loss dielectric waveguides, nanophotonic sensors, and on-chip photodetectors, as illustrated in Fig. 5a. vdW materials can play unique roles as building blocks for realizing waveguide-integrated nanophotonic biosensors. Enabling technologies towards this goal, in the context of mid-IR spectroscopy, including the development of widely-tunable quantum cascade lasers and optical parametric oscillators covering the entire mid-IR band, on-chip frequency combs, broadband fiber sources, imaging detectors, and maturing chalcogenide-based low-loss mid-IR waveguides^83^. Initially, using metal plasmonic elements, researchers have already addressed some of the challenges. Schwarz et al. demonstrated a compact mid-IR chemical sensing platform^84^, which monolithically integrates a bi-functional quantum cascade laser/detector coupled to a dielectric-loaded gold SPP waveguide (Fig. 5b). Waveguide-integrated SEIRA laser spectroscopy has been demonstrated with gold nanorod antennas pattered on a silicon photonic waveguide (Fig. 5c)^85^. Incorporation of vdW materials into waveguide geometries has numerous benefits to create biosensors with enhanced sensitivity and selectivity. Graphene^86^ and black phosphorus (Fig. 5d)^87^, have previously been demonstrated for use as modulators and detectors for optical communications applications, but similar structures have been shown to be well-suited for biosensing applications. 2D materials have the advantage that their optical response can be strongly affected by the chemical interactions, and they can readily be coupled to an adjacent waveguide optical platform. Numerous demonstrations in the literature have been made using this capability. Kim et al., demonstrated DNA and streptavidin sensing using a graphene-coated optical fiber via the SPR detection mechanism^88^. Alternatively, Yao et al., used a D-shaped polymer fiber Bragg grating coupled to a graphene layer to create a biochemical sensor. In this technique, the Bragg peak location was shifted in the presence of red blood cells and sub-ppm cellular concentrations were detected^89^. Kou et al.^90^ demonstrated a dopamine neurotransmitter sensor using graphene integrated on a silicon microring resonator geometry. The Si-based waveguide and ring resonator design give this sensor the advantage of a small sensing surface area of 30 μm^2^. The above demonstrations utilized sensors operating at telecom wavelengths. Greatly expanded detection of biologically relevant molecules could be achieved at mid-IR wavelengths. To this end, waveguide-integrated graphene chemical sensors for mid-IR operation have been proposed and studied theoretically^91,92^. Other platforms with potential for 2D-material mid-IR sensors include silicon-on-insulator (SOI) waveguide-integrated black phosphorous detectors^93^, and heterogeneously-integrated Si waveguides on mid-infrared compatible substrates, which could extend the detection limit out to 8 μm^94^. These advancements in miniaturization due to waveguide-mediated delivery of light onto the vdW sensor, could eventually eliminate the need for expensive and bulky FTIR spectrometers as well as laborious alignment of free-space optics via moving mechanical parts.

## Carbon nanotubes: 1D vdW for biophotonic sensors and single-molecule imaging

**Fig. 6:**
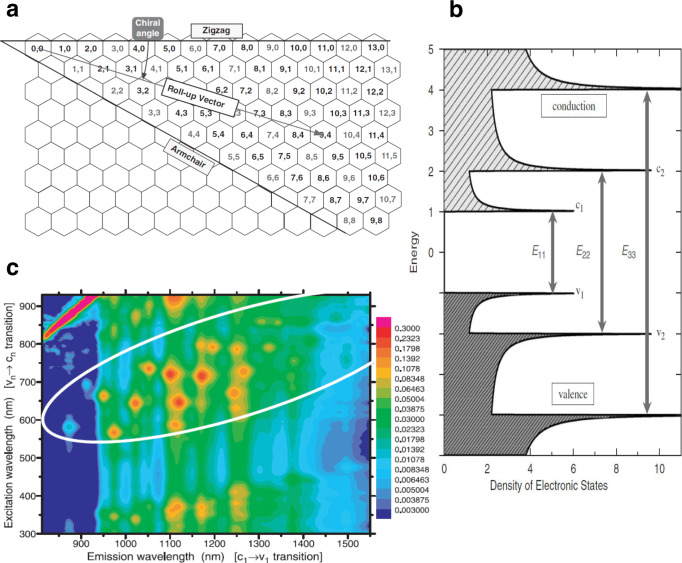
Properties of SWNTs. **a** Schematic illustrating the roll-up vector and chiral angle combinations informing SWNT chirality. **b** Band diagram illustrating the electronic transitions leading to SWNT fluorescence. Electrons are excited from *v*_2_ into *c*_2_, where they relax into *c*_1_. Upon recombination with a hole in *v*_1_, photons are emitted with an energy corresponding to the bandgap. **a**, **b** Reprinted from ref. ^98^ with permission. **c** Excitation-emission maps of individually dispersed SWNTS, with excitation wavelengths ranging between 500–900 nm and emission wavelength of 800–1600 nm. Reprinted from ref. ^97^ with permission.

While 2D vdW sensor substrates enable powerful in vitro sensing functions, 1D vdW SWNTs^95,96^ can address an entirely new set of problems—such as in vivo nanophotonic sensing and bioimaging. Shortly after the discovery of SWNTs’ near-infrared (NIR) fluorescence, this phenomenon was recognized as ideal for optical imaging and sensing^97^. The unique electronic properties of SWNTs that result in their bandgap fluorescence have been reviewed by Weisman^98^. Briefly, SWNTs can be thought of as single sheets of graphene rolled into cylindrical geometries. Their electronic—and thus photophysical—properties depend on the direction of rolling, which can be specified by lattice vectors *n* and *m* (Fig. 6a). Since specific lattice directions in graphene conduct, the rolling direction determines the SWNT bandgap and whether a specific (*n*, *m*) chiral species is metallic or semiconducting. The nanoscale-cylindrical shape of semi-conducting SWNTs confines electrons in one dimension along its circumference, leading to what is known as van Hove singularities in the electron density (Fig. 6b). The density of states in SWNTs scales as E^−1/2^ and diverges at the minima of electronic bands in 1D materials. Photoexcitation of SWNTs generates excitons, where the electron and hole are bound. The limited charge screening in quasi-1D structures allows strong electron-hole Coulomb interaction^99^ and the formation of stable excitons at room temperature. The exciton binding energy reaches 0.6 eV in 1-nm diameter SWNTs as determined by theoretical^100^ and experimental work^101^. The binding energy decreases as SWNT diameter increases. When the exciton is generated, it diffuses along the nanotube; for example, a diffusion length of 120 nm in 20 ps was reported for (6,5) SWNTs^102^. The diffusion length is limited by the presence of defects in the nanotube or the supporting substrate. Fluorescence occurs when the exciton relaxes back down to the band edge and emits a photon with energy equivalent to the exciton energy. SWNTs are commonly interrogated by exciting through a range of visible light in the range of the E_22_ transition, and their NIR E_11_ emission is then collected. Different semiconducting chirality species with different bandgaps/exciton energies can then be visualized using a 2D excitation-emission profile (Fig. 6c)^97^.

Due to intrinsically strong vdW interactions, SWNTs tend to stick together as bundles and do not naturally disperse in an aqueous solution or biological media. Covalent and non-covalent surface modifications of SWNTs are used to promote solubility. Covalent modification is achieved by chemical reactions on the nanotube surface to introduce carboxyl, amine, sulfhydryl, or other functional groups (Box 5)^103^. While offering a more stable suspension, covalent modification often alters the intrinsic mechanical strength and conductivity of SWNTs and increases exciton quenching. Thus, non-covalent modification is preferred to preserve their inherent electrical and optical properties. Non-covalent functionalization^104–106^ can be realized by entropy-driven interactions, such as hydrophobic interactions using surfactants such as sodium dodecyl sulfate (SDS)^107^, sodium dodecylbenzene sulfonate (SDBS)^108–110^, and sodium cholate (SC)^111,112^, and/or enthalpy-driven interactions such as π−π bonding, hydrogen bonding (CH−π, NH−π), or ionic interactions between the SWNT surface and dispersants such as aromatic polymers, cationic polymers, and block polymers.

**Fig. 7:**
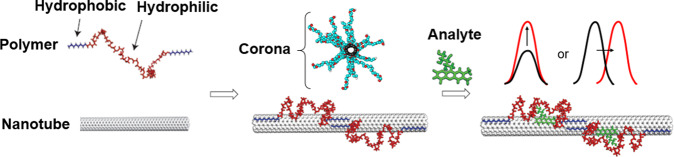
Schematic of the CoPhMoRe concept. SWNTs are wrapped with a polymer that forms a “corona” which modulates analyte binding. Upon recognition of a molecule, SWNT fluorescence changes in intensity or peak wavelength. Adapted with permission from ref. ^28^.

A new technique exploiting non-covalent interaction between SWNTs and polymers called corona phase molecular recognition (CoPhMoRe)^28^ has been developed to achieve selective recognition of specific molecules (Fig. 7). In CoPhMoRe, SWNTs are colloidally stabilized by amphiphilic polymers such as block copolymers or ssDNA, which form a 3D configuration called a “corona” in conjunction with the nanotube surface that can act as a synthetic molecular recognition site. Changes in the fluorescence of SWNTs upon molecular binding to the corona allow for the detection of such recognition events. Molecular recognition can influence SWNT fluorescence in several ways, including emission wavelength shifts due to solvatochromism and emission intensity changes caused by charge-transfer transition bleaching and exciton quenching. In solvatochromism, the introduction of analyte molecules changes the local dielectric environment of the SWNT by replacing solvent molecules, which modifies the optical transition energies^113^. In the electron transfer mechanism, the frequency at which the E_11_ fluorescence transition occurs is modulated by the interaction between the SWNT and the target molecule. The electrons that are otherwise allowed to release or prohibited from releasing photons through the E_11_ transition instead interact with the molecular orbitals of the analytes, which in turn changes the frequency of the transition^114,115^. Mechanistic understanding of SWNT fluorescence wavelength shift and intensity changes have enabled the development of SWNT-based fluorescent biosensors for the selective detection of a large array of analytes. To date, CoPhMoRe sensors have been fabricated to detect nitric oxide^115,116^, hydrogen peroxide^117^, vitamins^28^, neurotransmitters^118^, carbohydrates^119^, proteins^120,121^, and small-molecule drugs and steroids^28^. These measurements have been performed in a variety of environments, including buffer, cellular media, and living plant systems^27^. The design of corona phases for CoPhMoRe sensors draws upon a huge range of amphiphilic polymers, ranging from DNA sequences to phospholipids, to synthetic polymers. Lambert et al. used directed evolution of DNA sequences as a high-throughput strategy to quickly screen and optimize an SWNT wrapping that maximizes sensor fluorescent response^122^. Different approaches to interface biomolecules with metals and vdW materials are summarized in Box 5.

**Fig. 8:**
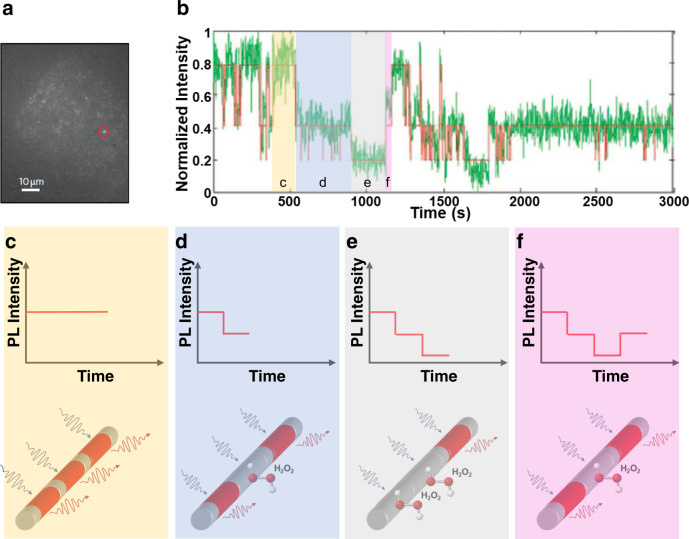
Single-molecule detection of H_2_O_2_ by stepwise quenching of individual SWNTs. **a** Near-infrared image of SWNTs under A431 cells. **b** Single-molecule tracking (green) performed by measuring the fluorescence of single sensors and monitoring step-changes in fluorescence, which can be fitted to a hidden Markov algorithm (red) to identify single binding and release events. Adapted from ref. ^117^ with permission. **c** A single nanotube (bottom) consists of multiple regions, where each state is either quenched or unquenched. The nanotube fluorescence intensity varies over time upon single-quencher molecular binding events. When unquenched, the fluorescence state at a particular region of the SWNT results in a nanotube fluorescence intensity value. **d** The adsorption of quenching H_2_O_2_ molecule results in a single-state quenching event, and a stepwise decrease in overall SWNT intensity. **e** Adsorption of another quenching molecule leads to a further decrease in overall intensity. **f** Desorption of H_2_O_2_ from the SWNT surface recovers the nanotube fluorescence with a stepwise increase in intensity.

Several groups have worked on improving the sensitivity of the CoPhMoRe method to image a single molecule. This is of great importance, as it enables detection of stochastic and dynamic interactions between biomolecules in bulk samples with high heterogeneity. Such measurements can be achieved by monitoring excitons that are induced by laser excitation and move along the sidewall of SWNTs with a characteristic diffusion length. When molecular quenchers bind to the corona/SWNT surface, the SWNT electronic structure is perturbed and excitons within a diffusion length of the adsorption site are quenched and undergo nonradiative recombination. Due to the excitonic nature of SWNT luminescence, the nanotubes do not photobleach, unlike traditional organic fluorophores. This allows for longer exposure times and higher laser fluences that can ultimately enable imaging of single molecules^123^. Cognet et al. have observed single proton and diazonium salt binding and release from SDBS-functionalized SWNTs by detecting stepwise changes in fluorescence^124^. This approach was extended by Zhang et al.^115^ to detect nitric oxide with SWNTs deposited onto functionalized glass sides, and by Jin et al.^125^ to detect H_2_O_2_ with SWNTs embedded in type 1 collagen thin films. During measurements, each SWNT serves as an individual optical probe, and individual stepwise changes in sensor states correspond to single-molecule binding or release events. The observed bulk SWNT fluorescence is a combination of these single-molecule interactions, and the bulk concentration is obtained through hidden Markov modeling^125^. The collagen-based SWNT sensors have also been applied to detect real-time H_2_O_2_ generation with high spatial resolution from individual live A431 human epidermal carcinoma cells stimulated with epidermal growth factor^117^ (Fig. 8). This approach can also be applied to understand small analyte efflux from microorganisms at the cellular level with fine spatial and temporal resolution. For example, Landry et al. immobilized RAP1 aptamer-anchored SWNT sensors in a microfluidic chamber and detected unlabeled RAP1 GTPase and HIV integrase selectively from various cell lines^126^. Similarly, Kruss et al. deposited a layer of SWNT sensors on a glass slide, cultured PC12 cells over them, and tracked dopamine efflux from single cells, correlating the curvature of the cell to neurotransmitter release^127^. At the tissue scale, SWNT sensors have been used to detect neurotransmitters in acute cultured brain slices^128^.

Box 5. Surface functionalization strategies for metals and vdW materials

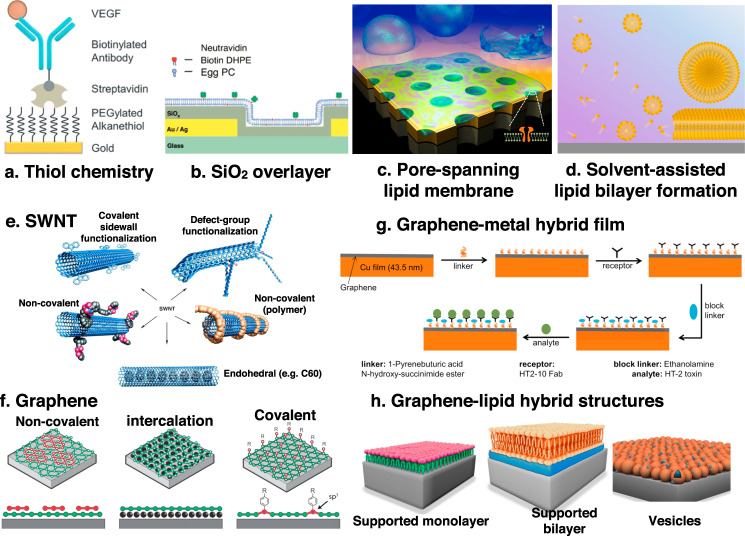
Nanophotonic biosensor performance is determined by the specific and robust binding of target analytes and the rejection of nonspecific binders. Thus, the surface chemistry is as important as the physical performance metrics discussed in Box 1. (a) Thiol chemistry with different terminal groups (e.g., carboxylate or amine) is widely used to covalently attach receptor molecules to gold or silver plasmonic surfaces. Reprinted from ref. ^170^ with permission. (b) Patterned nanophotonic structures typically utilize heterogeneous materials and interfaces (e.g., metals, dielectrics, or empty gaps). By conformally coating such structures with a thin SiO_2_ film via sputtering or ALD, one can make a homogeneous surface compatible with well-established silane chemistry or vesicle fusion to form a lipid bilayer membrane. Reprinted from ref. ^171^ with permission.

While ALD-grown oxides have been used for surface modification of metal/dielectric nanophotonic substrates, nucleation of ALD of oxides on graphene is difficult due to graphene’s hydrophobic nature and lack of out-of-plane bonds. Several strategies have been utilized to overcome this limitation, including using physisorbed ozone, hydrogen plasma functionalization, organic self-assembled monolayers, and evaporated metal or oxide seed layers. Such strategies have proven successful, provided that a pristine graphene surface can be achieved^172^.

For applications in membrane biology and drug discovery, it is desirable to interface nanophotonic sensors with highly fluidic lipid bilayer membranes or nanovesicles that can incorporate a rich group of membrane-bound receptors (e.g., membrane proteins, carbohydrates, or peptides). Membrane proteins are not easily manipulated in solution and do not fold properly outside the membrane, but researchers have used various schemes to address these challenges using a supported lipid bilayer (SLB)^173,174^, nanovesicles, or (c) a pore-spanning lipid bilayer (Reprinted from ref. ^175^ with permission). In general, SLBs are formed directly on a solid substrate with a thin layer of water (~1 nm) between the membrane and the substrate. It is critical that the sensor material (metal, dielectric, or vdW material) does not adversely affect the conformation or functionality of membrane-bound molecules. Recent work has combined SLBs, lipid nanovesicles, and synaptic vesicles for SEIRA sensing^47^. Importantly, lipid bilayer membranes can also help prevent nonspecific adsorption of proteins onto the sensor surface. (d) The recently-developed solvent-assisted lipid bilayer (SALB) method can be used to form lipid bilayer membranes directly on bare gold surfaces without a SiO_2_ layer. SALB can be useful for SPR, SERS, and SEIRA, and is compatible with some vdW materials. Reprinted from ref. ^176^ with permission. For substrates or receptors that are not amenable to these methods, other options are available, such as the use of membrane-derived particles as biomimetic receptors. Such cell-derived materials may be directly dispersed as nanoparticles on the surface, or physically inserted into patterned substrates via a ‘squeegee’ method^177^.

(e) Covalent and noncovalent functionalization schemes for SWNTs. Reprinted with permission from ref. ^103^. (f) The surface of graphene can be modified via different strategies, including non-covalent functionalization (π-stacking), intercalation of molecules between graphene and a substrate, and covalent chemical modification of graphene. Image credit: Kim Daasbjerg. (g) Illustration of surface modification and blocking strategies for graphene-coated Cu films for SPR biosensing. Reprinted with permission from ref. ^39^.

(h) SLB formation on graphene is not as clearly understood as for gold or SiO_2_ surfaces. Graphene is normally hydrophobic in its pristine state, which hinders SLB formation. Interestingly, graphene is also known to exhibit “wetting transparency” to the underlying substrate^178^. For example, an SLB can be formed via vesicle rupture on graphene on top of a hydrophilic silica surface. In contrast, graphene on gold, which is hydrophobic, leads to the formation of an intact vesicle layer. Indeed, recent work showed that a lipid monolayer, bilayer, or layer of intact vesicles could be formed on graphene depending on the surface treatment^179^. Biological interfacing of vdW materials with soft lipid membrane interfaces can add unique options to augment the functionality of nanophotonic biosensors, and further research is highly desirable.

## Towards SWNT-based POC diagnostics and in vivo applications

SWNT-based sensors have also been developed to detect disease biomarkers at low concentrations, with potential application to POC diagnostics, enabling timely treatment at a reduced cost^129,130^. Label-free optical SWNT sensors are preferable to traditional POC detection methods such as ELISA in that they do not require additional processing steps after sample collection and allow transient data collection^131^. One example is a fluorescent SWNT-based microarray for detecting real-time protein-protein interactions^132^. Here, chitosan-wrapped SWNTs are conjugated to nitrilotriacetic acid (NTA) groups that can chelate Ni^2+^. The NTA-Ni^2+^ complex then binds to a His-tag on the target proteins, and the binding event is detected by the reduction of SWNT fluorescence. This platform has been further engineered to monitor 1156 capture protein-protein analyte interactions in a signaling network important in apoptosis. Using a different molecular chelator, Dong et al.^133^ conjugated immunoglobulin-binding proteins to SWNT and detected IgG, IgM, IgG2a, and IgD. Capture protein-modified sensors can also be printed onto a microarray that examines cross-reactivity, competitive, and nonspecific binding of analyte mixtures^134^. Such optical nanosensors have great potential to advance real-time, multiplexed biomarker detection for disease diagnostics.

The unique optoelectronic properties of SWNTs are also ideal for in vivo biosensing applications. SWNTs fluoresce within the NIR window (900–1500 nm), where scattering is reduced and tissue absorption is minimal, allowing SWNT fluorescence to penetrate through deeper layers of tissue relative to visible light^135,136^. A central question that limits biological applications relates to the biocompatibility of SWNTs. Their toxicity has been evaluated in vitro, with a variety of factors including chirality, length^137^, synthesis method^138^, SWNT wrapping^139,140^ and cell type^125,140,141^, all affecting biocompatibility. SWNTs are a broad class of materials with varying dimensions, functionalization, and formulation, and since many aspects of a given preparation can affect biological responses^142^, it is important to evaluate toxicity on a case-by-case basis. In addition, response in cell culture might not always reflect in vivo tissue effects, where cell populations are far more heterogeneous, and materials might also be modified once introduced into the body. Thus, it is crucial to test SWNT sensors in vivo, under conditions reflecting the intended route of administration, site of action, and dose.

SWNTs have been widely applied in animal models. Iverson et al. delivered ss(AAAT)_7_-wrapped SWNTs via tail vein injection to detect transient inflammation, using nitric oxide as a signaling marker in mouse liver. The authors demonstrated long-term nitric oxide monitoring by subcutaneous implantation of alginate hydrogel-encapsulated SWNT sensors. The sensor fluorescence signal remained stable for over 400 days, and histological analysis of the implantation sites showed negligible adverse response^116^. Harvey et al. loaded DNA-wrapped SWNT sensors into a semipermeable membrane and implanted these into the intraperitoneal (IP) space in mice. The sensors successfully detected oligonucleotide hybridization in vivo, and the mice exhibited no obvious behavior changes^143^. SWNT sensors have also been used to detect lipid concentrations in vivo in the mouse liver^144^. Galassi et al. injected DNA-wrapped SWNTs through the tail vein in mice, which localized to the liver^144^. These DNA SWNTs showed a wavelength shift in response to changing lipid concentrations, and once the mice were administered a high-calorie diet, the resulting fat accumulation within the liver could be detected and used to determine the onset of fatty liver disease. SWNTs implanted in the liver maintained the ability to shift wavelength in response to their analyte over a period of three months, showing the stability of such sensors.

Williams et al. encapsulated antibody-conjugated/DNA-wrapped SWNTs into a dialysis bag and implanted them into the IP space of mice^145^. These sensors detected the ovarian cancer biomarker human epididymis protein 4 (HE4) from both exogenous administration and endogenous production. DNA-wrapped SWNTs encapsulated in poly(ethylene glycol) diacrylate hydrogels have also been used in various marine organisms for biologging purposes, with minimal changes in tissue architecture for catfish, shark, and eel as detected by high-resolution ultrasound^146^. These examples show that SWNT sensors of various designs have the potential to function safely in vivo.

## Conclusions and future perspectives

We have explored nanophotonic biosensing technologies enabled by low-dimensional vdW materials. With continually improving large-scale synthesis methods for vdW materials, high-resolution lithography, and ultrasensitive detection schemes, it may soon be possible to harness these versatile materials for practical POC biosensing applications. Further in the future, this technology could grow to enable previously inaccessible applications such as label-free monitoring of protein conformational dynamics, flexible and wearable biosensors, and in vivo nanophotonic biosensors. Such real-time in vivo sensors could potentially provide quantitative measurements of biological networks, and in humans, could allow prolonged data collection for early detection of disease, or even be coupled to drug delivery devices to enable closed-loop disease control—analogous to the ‘artificial pancreas’ systems that have been developed for continuous glucose control in diabetes. We also expect vdW materials to benefit other functions beyond conventional sensing that will still contribute to better biosensors, such as harnessing vdW materials for on-chip liquid manipulation (e.g., liquid sealing, or nanofluidics), high-gradient-field particle trapping, or building waveguide-integrated light sources and photodetectors.

Research into graphene and 2D quantum materials for SEIRA sensing is still in its infancy. In the near future, we will likely witness innovations combining high-quality 2D materials with smart choices of substrates and device configurations. These efforts will undoubtedly improve the performance and expand the versatility of SEIRA-based sensors for a wide range of applications.

Another exciting strategy for accessing physical and chemical information entails the physical confinement of individual analyte molecules in a configuration with lower degrees of freedom. One such detection modality is based on the confinement of molecules into structures fabricated in the matrix of the 2D material, such as nanoscale apertures or nanopores (Supplementary Note 1).

The library of vdW materials and heterostructures is rapidly expanding to offer customizable choices for fabricating unique nanophotonic biosensors. For SEIRA and other biosensing applications, we envision that hybrid substrates integrating graphene, polar materials, vdW heterostructures, metal antennas, high-Q dielectric metasurfaces^52,147^, and low-loss dielectric waveguides will present unique opportunities for future innovation. Two-dimensional TMDCs have demonstrated strong exciton resonances in optical spectra^24,148^, and recent work has shown that the presence of molecules can transform dark states into bright excitons, potentially enabling unambiguous detection of the adsorbed molecules. Excitons in 2D TMDCs can also be tuned by various approaches^149^. In addition, integration with birefringent and chiral metasurfaces e.g., chiral plasmons or twisted graphene layers^150,151^ or valley-dependent chiral coupling in 2D TMDCs^152^ for measuring chiro-optic response could advance enantioselective biochemical sensing applications^153^.

While this review focused on nanophotonic sensors, electronic and electrochemical sensors based on vdW materials have also been extensively investigated^154,155^. Ultimately, vdW materials may empower researchers to realize one of the overarching ambitions of nanophotonics—seamless integration of photonics and electronics in the field of biosensing.

## Supplementary information

Supplementary Information
