# Supplementary material for: Nanophotonic biosensors harnessing van der Waals materials
Source: Nat Commun. 2021 Jun 22;12:3824. doi: 10.1038/s41467-021-23564-4 (PMC8219843; doi:10.1038/s41467-021-23564-4)
Supplement: Supplementary file 1 — Supplementary Information [file 41467_2021_23564_MOESM1_ESM.pdf]

Supporting Information

Nanophotonic Biosensors Harnessing van der Waals Materials

Sang-Hyun Oh[1*], Hatice Altug[2*], Xiaojia Jin[3], Tony Low[1], Steven J. Koester[1], Aleksandar P. Ivanov[4], Joshua B. Edel[4], Phaedon Avouris[5], and Michael Strano[3*]

[1] Department of Electrical & Computer Engineering, University of Minnesota, Minneapolis, MN, USA

[2] Institute of Bioengineering, École Polytechnique Fédérale de Lausanne (EPFL), Lausanne 1015, Switzerland

[3] Department of Chemical Engineering, Massachusetts Institute of Technology, Cambridge, MA, USA

[4] Department of Chemistry, Imperial College London, South Kensington Campus, London, UK

[5] IBM T. J. Watson Research Center, Yorktown Heights, New York, NY, USA

*Address correspondence to sang@umn.edu, hatice.altug@epfl.ch, strano@mit.edu

2D vdW materials for nanopore sensing

Beyond electromagnetic field confinement in 2D materials, one of the most exciting applications to access molecular information is the ability to physically confine individual analyte molecules in a configuration with lower degrees of freedom. Depending on the 2D material and experiment, information about the physical and chemical properties of the analyte can be extracted, often at the single-molecule level. An emerging detection modality is based on the confinement of molecules into structures fabricated in the matrix of a 2D material. One very promising strategy is in the incorporation of nanoscale apertures or nanopores in such materials for applications in biomolecular sensing[1]. Typically, the diameter of the nanopore is less than 10 nm, closely matching the dimension of individual molecules or within an order of magnitude of the screening length of individual ions. The selection of the correct membrane material is highly significant in ensuring efficient molecular transport through the nanopore. With this in mind, there has been a drive to design membranes from 2D materials, which offers unprecedented advantaged over more conventional 3D materials.

The principles of conventional nanopore-based molecular sensing are straightforward; briefly, a constant voltage bias is applied across the nanopore that connects two reservoirs filled with electrolyte separated by a non-permeable and insulating membrane. Although 2D materials can be only a single atomic layer thick, they are often excellent ionic insulators, and the applied bias causes a steady-state ionic current to flow through the nanopore and as the resistance of the nanopore is greater than that of the electrolyte, majority of the potential drop occurs at or close to the nanopore. Ions and any other charged molecules are therefore transported (or translocated) though the nanopore as a result of the generated potential gradient. When a molecule enters the nanopore, the ionic current more often than not decreases, which results in a current blockade; however, current enhancement can also be obtained depending on the analyte properties, material properties of the membrane, and electrolytic conditions. The amplitude, duration, and in some cases the shape of the current change can reveal valuable information of the molecule being probed, including size, charge, lengths, and affinity amongst others.[2] Importantly these measurements are obtained for each translocated molecule, instead of measuring the average behaviour of an ensemble of molecules.

Although applications using nanopores are rapidly growing, they are not suitable for use in all circumstances. Both the nanopore diameter and membrane thickness are crucial for maximising the signal-to-noise (S/N) levels and require careful optimisation based on the target analyte. As a general rule, the smaller the nanopore diameter, the greater the S/N. Fortunately, nowadays there is an arsenal of established techniques for making nanoscale pores in solid-state materials, including transmission electron microscopy,[3,4] ion milling and focused-ion-beam (FIB) microscopy[5,6,7] electron-beam lithography[8,9,10] dielectric breakdown,[11] laser etching[12]. For a detailed discussion of these methods, the reader is referred to alternative reviews.[13] Often single-digit-nanometer and even sub-nanometer pores can be achieved with high reproducibility. This flexibility in tuning nanopore dimensions has been arguably the single most crucial advantage of solid-state nanopores over their biological counterparts where the nanopore dimension is typically fixed depending on the protein being used.

Much like the nanopore diameter, the membrane thickness also plays a large role in maximising the signal-to-noise, where to a first approximation, the paradigm, less is more, is most certainly true. However, due to advances in materials science, it has recently only been possible to reliably fabricate nanopores within solid-state materials with sub-10 nm thickness, let alone 2D materials. The membrane thickness can be controlled using a bottom-up or top-down approach or a combination of both. In terms of bottom-up approaches, ultrathin membrane materials have been fabricated using ALD and chemical vapour deposition (CVD). Traditionally materials such as SiO_2, TiO_2, HfO_2, Al_2O_3 and graphene or even multiple layers of different materials can be deposited over a supporting dielectric structure usually made of SiN_x or SiO_2, fabricated on a Si wafer. An alternative fabrication strategy is the exfoliation or CVD growth of 2D materials such as graphene, MoS_2, hBN into nanosheets, followed by their transfer onto the supporting structure.[14,15,16,17,18,19,20]

Alternatively, top-down approaches such as local thinning of existing solid-state membranes have also been documented. In most cases, silicon nitride (SiN_x), which is a traditionally used as the stoichiometry can be

modified to tune the Si to N ratio and to form high-aspect ratio membranes with low- tensile stress and high mechanical robustness. Local sections of the SiN membrane can be selectively etched, either using dry etching[21], electron or ion-beam irradiation,[22,23] or by laser-assisted photothermal etching[24]. Nanopores can then be formed using conventional fabrication methods (usually using electron/ion microscope, dielectric breakdown, or laser etching), with the significant advantage of having a well-defined pore shape and thickness approaching atomic dimensions.

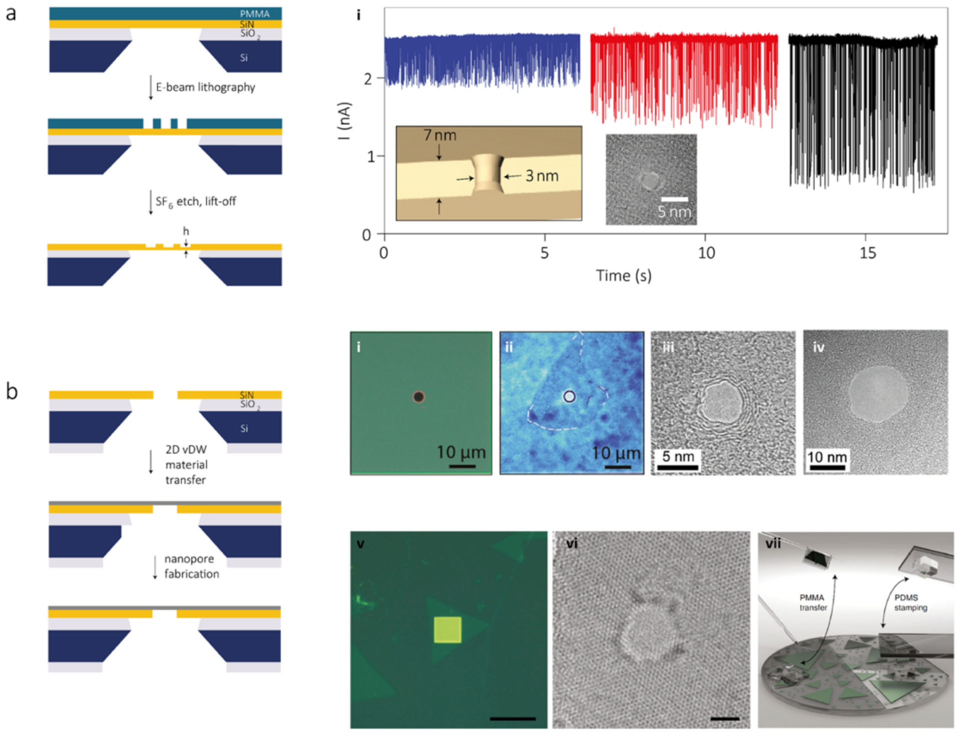

Figure S1 | Strategies for making 2D membranes. a) local thinning. In this example, a SiN membrane is locally thinned using SF_6 to locally etch the membrane around the nanopore. (i) The increase in signal to noise could clearly be seen for thinner membranes as is shown for the translocation of 3 kbp dsDNA, adapted from 21. b) transfer of 2D vdW material. (i-iv) Graphene membrane including optical micrographs depicting the transfer of graphene on Si/SiO_2. The nanopores were made using a highly focussed electron beam of a transmission electron microscope, adapted from 17. (v-vii) Overview of the fabrication process for making MoS_2 nanopores including an optical micrograph of the transferred material on a SiN membrane (scale bar 20 µm) and a TEM image of a drilled nanopore (scale bar 2 nm), adapted from 31.

A key advantage of utilising nanopores in 2D vdW materials is in the substantial increase in spatial resolution and S/N, due to the shorter length over which the ionic current signal is probed and the decrease of the nanopore resistance. These advantages can lead to enhanced sensitivity to small molecular differences; however, it is essential to highlight that to obtain transformative information at the submolecular level, the temporal resolution needs to be improved as well, or strategies for slowing down molecular transport need to be implemented. Hybrid 2D membrane materials can be used to tune interactions with specific analytes. For example, nucleic acid translocation can be slowed down due to increased electrostatic interaction with Al_2O_3 or ZnO membranes,[25,26] or due to physicochemical interaction in HfO_2 membranes[27]. Furthermore, single-stranded DNA has also been shown to be slowed down in graphene due to non-specific adsorption. Also, the unique electronic properties of that 2D materials can exhibit additional detection modalities. For example, the possibility of using in-plane electronic conductivity in graphene membranes or semiconducting MoS_2 membranes can enable additional channels for molecules detection or manipulation.

It should be noted that one of the challenges of using 2D membrane materials such as graphene is the increased hydrophobicity, leading to the unwanted adsorption of analytes such as DNA onto the membrane surface.[28] However, the strong hydrophobic interaction between DNA and graphene can be minimised using surface treatments of graphene or 2D vdW materials that pose enriched hydrophilic surface sites such as MoS_2.[29,30,31]

References

[1] Shi, W.Q., Friedman, A.K., Baker, L.A. Nanopore Sensing. *Anal. Chem.* **89**, 157-188 (2017).

[2] Miles, B.N., Ivanov, A.P., Wilson, K.A, Dogan, F., Japrung, D., Edel, J.B. Single molecule sensing with solid-state nanopores: novel materials, methods, and applications. *Chem. Soc. Rev.* **42**, 15-28 (2013).

[3] Storm, A.J., Chen, J.H., Ling, X.S., Zandbergen, H.W., Dekker, C. Fabrication of solid-state nanopores with single-nanometre precision. *Nat. Mater.* **2**, 537-540 (2003).

[4] Kim, M.J., Wanunu, M., Bell, D.C., Meller A. Rapid fabrication of uniformly sized nanopores and nanopore arrays for parallel DNA analysis. *Adv. Mater.* **18**, 3149-3153 (2006).

[5] Li, J., Stein, D., McMullan, C., Branton, D., Aziz, M.J., Golovchenko, J.A. Ion-beam sculpting at nanometre length scales. *Nature* **412**, 166-169 (2001).

[6] Gierak, J.*, et al.* Sub-5 nm FIB direct patterning of nanodevices. *Microelectron. Eng.* **84**, 779-783 (2007).

[7] Yang, J.J.*, et al.* Rapid and precise scanning helium ion microscope milling of solid-state nanopores for biomolecule detection. *Nanotechnology* **22**, 285310 (2011).

[8] Han, A.P.*, et al.* Sensing protein molecules using nanofabricated pores. *Appl. Phys. Lett.* **88**, 093901 (2006).

[9] Wei, R.S., Pedone, D., Zurner, A., Doblinger, M., Rant, U. Fabrication of Metallized Nanopores in Silicon Nitride Membranes for Single-Molecule Sensing. *Small* **6**, 1406-1414 (2010).

[10] Verschueren, D.V., Yang, W., Dekker, C. Lithography-based fabrication of nanopore arrays in freestanding SiN and graphene membranes. *Nanotechnology* **29**, 145302 (2018).

[11] Kwok, H., Briggs, K., Tabard-Cossa, V. Nanopore Fabrication by Controlled Dielectric Breakdown. *Plos One* **9**, e92880 (2014).

[12] Gilboa, T., Zrehen, A., Girsault, A., Meller, A. Optically-Monitored Nanopore Fabrication Using a Focused Laser Beam. *Sci. Rep.* **8**, 9765 (2018).

[13] Lee, K.; Park, K.-B.; Kim, H.-J.; Yu, J.-S.; Chae, H.; Kim, H.-M.; Kim, K.-B. Recent Progress in Solid-State Nanopores. *Adv. Mater.* **30**, 1704680 (2018).

[14] Liu, S.*, et al.* Boron Nitride Nanopores: Highly Sensitive DNA Single-Molecule Detectors. *Adv. Mater.* **25**, 4549-4554 (2013).

[15] Garaj, S., Hubbard, W., Reina, A., Kong, J., Branton, D., Golovchenko, JA. Graphene as a subnanometre trans-electrode membrane. *Nature* **467**, 190-193 (2010).

[16] Merchant, C.A.*, et al.* DNA Translocation through Graphene Nanopores. *Nano Lett* **10**, 2915-2921 (2010).

[17] Schneider, G.F.*, et al.* DNA Translocation through Graphene Nanopores. *Nano Lett* **10**, 3163-3167 (2010).

[18] Zhou, Z.*, et al.* DNA Translocation through Hydrophilic Nanopore in Hexagonal Boron Nitride. *Sci. Rep.* **3**, 3287 (2013).

[19] Park, K.B.*, et al.* Noise and sensitivity characteristics of solid-state nanopores with a boron nitride 2-D membrane on a pyrex substrate. *Nanoscale* **8**, 5755-5763 (2016).

[20] Waduge, P.*, et al.* Direct and Scalable Deposition of Atomically Thin Low-Noise MoS_2 Membranes on Apertures. *ACS Nano* **9**, 7352-7359 (2015).

[21] Wanunu, M., Dadosh, T., Ray, V., Jin, J.M., McReynolds, L., Drndic, M. Rapid electronic detection of probe-specific microRNAs using thin nanopore sensors. *Nat. Nanotechnol.* **5**, 807-814 (2010).

[22] Sawafta, F., Carlsen, A.T., Hall, A.R. Membrane Thickness Dependence of Nanopore Formation with a Focused Helium Ion Beam. *Sensors* **14**, 8150-8161 (2014).

[23] Rodriguez-Manzo, J.A., Puster, M., Nicolai, A., Meunier, V., Drndic, M. DNA Translocation in Nanometer Thick Silicon Nanopores. *ACS Nano* **9**, 6555-6564 (2015).

[24] Yamazaki, H., Hu, R., Zhao, Q., Wanunu, M. Photothermally Assisted Thinning of Silicon Nitride Membranes for Ultrathin Asymmetric Nanopores. *ACS Nano* **12**, 12472-12481 (2018).

[25] Venkatesan, B.M., Shah, A.B., Zuo, J.M., Bashir, R. DNA Sensing Using Nanocrystalline Surface-Enhanced Al2O3 Nanopore Sensors. *Adv. Funct. Mater.* **20**, 1266-1275 (2010).

[26] Park, K.B., *et al.* Highly reliable and low-noise solid-state nanopores with an atomic layer deposited ZnO membrane on a quartz substrate. *Nanoscale* **9**, 18772-18780 (2017).

[27] Larkin, J., Henley, R., Bell, D.C., Cohen-Karni, T., Rosenstein, J.K., Wanunu, M. Slow DNA Transport through Nanopores in Hafnium Oxide Membranes. *ACS Nano* **7**, 10121-10128 (2013).

[28] Schneider, G.F., *et al.* Tailoring the hydrophobicity of graphene for its use as nanopores for DNA translocation. *Nat. Commun.* **4**, 2619 (2013).

[29] Liu, K., Feng, J.D., Kis, A., Radenovic, A. Atomically Thin Molybdenum Disulfide Nanopores with High Sensitivity for DNA Trans location. *ACS Nano* **8**, 2504-2511 (2014).

[30] Farimani, A.B., Min, K., Aluru, N.R. DNA Base Detection Using a Single-Layer MoS_2. *ACS Nano* **8**, 7914-7922 (2014).

[31] Graf, M., *et al.* Fabrication and practical applications of molybdenum disulfide nanopores. *Nat Protoc* **14**, 1130-1168 (2019).
